# The multi-target protective effects of quercetin in cerebrovascular diseases: a dietary strategy for endothelial repair and neuroprotection

**DOI:** 10.3389/fnut.2026.1775964

**Published:** 2026-02-18

**Authors:** Tong Lu, Junjie Gao, Ping Zhu, Ruoxue Cao, Kai Ye

**Affiliations:** 1 Department of Clinical Laboratory, The Second Affiliated Hospital of Wannan Medical College, Wuhu, China; 2 Department of Laboratory Medicine, The Second People’s Hospital of Lianyungang, Lianyungang, China

**Keywords:** bioavailability, cerebrovascular disease, neuroprotection, quercetin, vascular endothelial repair

## Abstract

Cerebrovascular diseases, including ischemic stroke and vascular cognitive impairment, represent a significant global health challenge due to the paucity of effective treatment options. Quercetin, a dietary flavonol, has emerged as a promising multi-target neuroprotective compound. This review elucidates the core mechanisms by which quercetin achieves vascular repair and neuroprotection in cerebrovascular diseases through synergistic regulation of multiple signaling pathways and explores strategies to bridge the gap between dietary intake and clinical application. At the vascular level, quercetin enhances antioxidant defense by activating the nuclear factor E2-related factor 2/heme oxygenase-1 axis, inhibits the Toll-like receptor 4/nuclear factor-κB pathway and NOD-like receptor protein 3 inflammasome, and maintains blood–brain barrier integrity by inhibiting matrix metalloproteinase-9 and upregulating tight junction proteins via the Wnt/*β*-catenin signaling pathway. At the neural level, quercetin inhibits apoptosis through the brain-derived neurotrophic factor-PI3K/Akt pathway, promotes M1-to-M2 microglial polarization to control neuroinflammation, and enhances synaptic plasticity. Additionally, quercetin exerts beneficial effects on mitochondrial protection and calcium homeostasis regulation. However, quercetin currently faces significant barriers to bioavailability, including low oral bioavailability and limited ability to cross the blood–brain barrier. Emerging nanotechnology-based delivery systems, including liposomes, exosomes, and reactive oxygen species-responsive nanoparticles, can enhance brain targeting and bioavailability. Future research should prioritize promoting dietary patterns rich in flavonoid compounds while developing advanced formulations validated through rigorous clinical trials. This approach will help clarify effective dosages, safety profiles, and clinical endpoint benefits across different stages of cerebrovascular disease. In summary, quercetin represents a promising candidate for cerebrovascular disease intervention; however, technological innovations are urgently needed to overcome bioavailability limitations and generate conclusive clinical evidence.

## 1 Introduction

Cerebrovascular diseases represent a significant challenge to global public health (1). Ischemic stroke constitutes a major cause of both mortality and permanent disability in adults (2–4). The pathophysiological process is complex, involving multiple cascading reactions, including energy depletion, excitotoxicity, oxidative stress, inflammatory responses, blood–brain barrier (BBB) disruption, and neuronal death (5–7). Currently, standard treatments for acute ischemic stroke comprise intravenous thrombolysis (e.g., rt-PA) and mechanical thrombectomy (8, 9). However, both approaches are subject to strict time-window limitations, benefiting only a minority of patients (10–13). Furthermore, stroke survivors frequently experience residual motor, sensory, and cognitive impairments that severely compromise quality of life (14–16). Additionally, there remains a paucity of effective pharmaceuticals to promote neurological recovery. Vascular cognitive impairment (VCI), another prevalent cerebrovascular disorder, exhibits rising incidence with population aging and lacks specific therapeutic interventions. VCI encompasses a broad spectrum of cognitive impairments, ranging from mild cognitive impairment to vascular dementia caused by ischemic or hemorrhagic stroke, as well as vascular factors acting alone or in combination with neurodegenerative pathology, including Alzheimer’s disease and related dementias (17–21). The cumulative or synergistic effects of neurodegenerative and cerebrovascular pathology represent the most significant contributors to dementia (22, 23).

Given the limitations of existing treatments, preventive medicine and complementary therapeutic strategies are gaining increasing attention, particularly bioactive compounds derived from natural sources with multi-target regulatory activities. Dietary intervention, as a safe, economical, and easily implementable health strategy, demonstrates significant potential in the prevention and management of chronic diseases (24–26). Quercetin, a dietary polyphenol belonging to the flavonol subclass, is the predominant flavonol in Western diets, accounting for 60–75% of total flavonol intake (27, 28). Of all the major flavonoid classes, quercetin typically makes up 10–20% of total dietary flavonoid consumption. Mean daily intake ranges from 10 to 16 mg/day in Western populations, with substantial geographical and cultural variation based on dietary patterns (29). This compound primarily functions by binding to sugar moieties, thereby forming the backbone structure of flavonoid compounds, including rutin, hesperidin, naringin, and citrusin. Its distinctive chemical configuration confers the ability to scavenge free radicals and chelate metal ions with high efficiency (30). Due to its abundant presence in plant-based foods such as onions, apples, berries, tea leaves, and legumes, along with its broad pharmacological activities—including antioxidant, cardiovascular protective, neuroprotective, antiviral, anticancer, antibacterial, anti-inflammatory, hepatoprotective, and anti-obesity effects—quercetin has become a research focus for preventing lifestyle-related diseases (31).

In recent years, substantial preclinical research has demonstrated the significant protective effects of quercetin across various animal models of cerebrovascular diseases (32). Quercetin has been shown to reduce infarct volume, alleviate neuroinflammation, and improve neurological deficits (33). Furthermore, it modulates multiple disease-progression-related signaling pathways at the molecular level (34). The mechanism of action of this compound extends beyond simple antioxidant effects, forming a synergistic network of “multiple targets and pathways” spanning from vascular protection to neuroprotection (35, 36). However, despite substantial evidence supporting the efficacy of quercetin in cerebrovascular diseases, its clinical translation remains slow due to low oral bioavailability and the difficulty of free quercetin effectively penetrating brain tissue. The present review aims to systematically elucidate quercetin’s multi-target protective mechanisms in cerebrovascular diseases, thoroughly analyze core challenges from dietary intake to clinical application, and explore future dietary strategies and innovative technological pathways designed to bridge the “translation gap.” The objective is to provide scientific rationale and direction for the contemporary utilization of this natural molecule.

## 2 The core protective mechanism of quercetin: a multi-target synergistic interaction network

**Figure 1:**
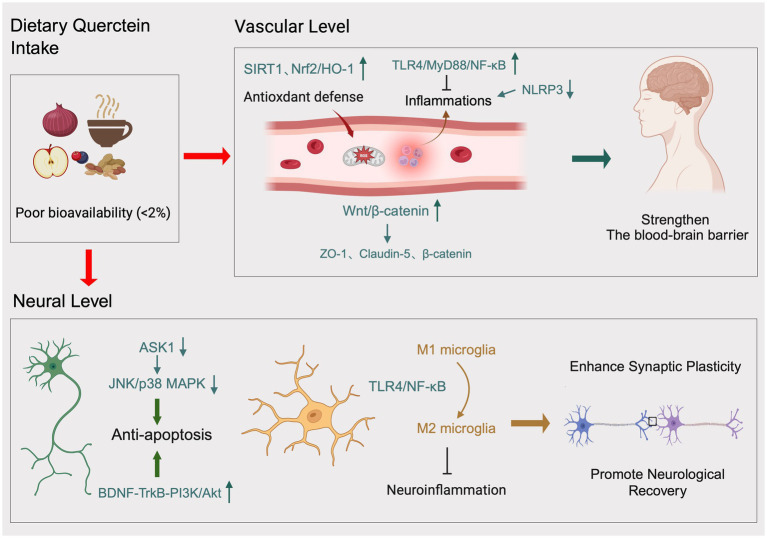
Dietary quercetin: vascular and neuroprotective mechanisms mediated by antioxidant, anti-inflammatory, anti-apoptotic, and synaptic plasticity-enhancing pathways.

The neuroprotective effects of quercetin are not attributable to a single mechanism but rather result from a complex and interconnected molecular network (37–42). This network can be summarized at two core levels in Figure 1: first, direct protection of the cerebral vascular system, focusing on restoring endothelial function and maintaining blood–brain barrier integrity; second, direct protection of neurons, including inhibiting cell death, regulating neuroinflammation, maintaining calcium homeostasis, protecting mitochondria, and promoting functional remodeling (43, 44).

### 2.1 Repairing the vascular endothelium and maintaining the BBB integrity

Cerebral vascular endothelial cells constitute the structural and functional core of the BBB. In cerebrovascular events such as ischemic stroke, endothelial cell injury represents a critical early trigger for subsequent pathological cascades. Endothelial dysfunction increases BBB permeability, allowing harmful substances and inflammatory cells from the bloodstream to infiltrate the brain parenchyma, inducing cerebral edema and exacerbating neuronal damage (40). Quercetin has been shown to enhance vascular defense mechanisms through multiple synergistic pathways (44).

#### 2.1.1 Enhancement of the antioxidant defense system

Oxidative stress is a hallmark feature of ischemia/reperfusion injury. Excessive reactive oxygen species directly attack endothelial cell membranes, proteins, and DNA, leading to cellular dysfunction and death (45, 46). Quercetin’s multifaceted role as an antioxidant is well-documented, and its ability to enhance intrinsic antioxidant defense mechanisms is particularly noteworthy (47–49). Among these mechanisms, the nuclear factor E2-related factor 2 (Nrf2) signaling pathway plays a central role (50). Under physiological conditions, Nrf2 binds to the cytoplasmic Keap1 protein, resulting in Nrf2 degradation (51, 52). Under oxidative stress conditions, quercetin facilitates the dissociation of Nrf2 from Keap1, enabling activated Nrf2 to translocate into the nucleus (53). There, it binds to antioxidant response elements, initiating transcription of downstream antioxidant and detoxification enzymes, including heme oxygenase-1 (HO-1) and glutathione peroxidase. Multiple animal models of ischemic stroke have confirmed that quercetin significantly upregulates Nrf2 and HO-1 expression in ischemic brain regions while reducing oxidative stress markers, effectively protecting endothelial cells and maintaining BBB integrity (54, 55). Furthermore, Sirtuin 1 (SIRT1) plays a role in this process. SIRT1, an NAD^+^-dependent deacetylase, activates Nrf2 through deacetylation modification, establishing the SIRT1/Nrf2/HO-1 signaling axis (56). This axis functions synergistically to resist oxidative damage and protect the BBB and neurons (57).

#### 2.1.2 Suppression of endothelial inflammatory responses

Inflammation is a significant contributor to ischemic brain injury (58, 59). Damaged endothelial cells overexpress adhesion molecules and release large amounts of proinflammatory cytokines, recruiting inflammatory cells such as neutrophils to infiltrate brain tissue and exacerbate secondary damage (60). Quercetin demonstrates significant anti-inflammatory properties, targeting a wide range of inflammatory pathways with precision (61, 62).

Firstly, quercetin effectively inhibits the Toll-like receptor 4 (TLR4) signaling pathway (63, 64). TLR4 is a pivotal receptor that recognizes damage-associated molecular patterns (65). Activation of this pathway, initiated by the downstream adaptor protein MyD88, results in nuclear factor-κB (NF-κB) activation and nuclear translocation (61, 63). NF-κB, a core transcription factor in the inflammatory response, plays a crucial role in transcribing pro-inflammatory genes, including tumor necrosis factor-*α* (TNF-α), interleukin-1β (IL-1β), and interleukin-6 (IL-6). Research has demonstrated that quercetin substantially inhibits TLR4/MyD88/NF-κB pathway activation in ischemic brain tissue, reducing proinflammatory factor production and alleviating endothelial inflammation (64, 66, 67).

Furthermore, quercetin directly targets and inhibits NLRP3 inflammasome activation. The NLRP3 inflammasome is a cytoplasmic multiprotein complex that assembles and activates upon sensing danger signals, such as reactive oxygen species. It cleaves pro-Caspase-1 into active Caspase-1, which subsequently processes pro-IL-1β and pro-IL-18 into mature proinflammatory cytokines IL-1β and IL-18 (68). By inhibiting NLRP3 inflammasome assembly and activation, quercetin effectively impedes the propagation of this inflammatory signal at its origin. Additionally, studies have demonstrated that quercetin mitigates endoplasmic reticulum stress by modulating stress-related protein expression, thereby safeguarding endothelial cells from stress-induced apoptosis (69, 70).

#### 2.1.3 Stabilization of BBB structural foundations

The physical barrier integrity of the BBB is contingent upon tight junction and adhesion junction proteins between endothelial cells (71–73). Ischemia/reperfusion injury leads to degradation and redistribution of these junction proteins, thereby compromising barrier integrity (74–76). Quercetin reinforces this barrier through multiple mechanisms (77, 78).

Firstly, quercetin inhibits the activity of matrix metalloproteinases, particularly matrix metalloproteinase-9 (MMP-9) (79). MMP-9 is significantly activated after ischemia and serves as a key enzyme that degrades tight junction proteins (such as Claudin-5 and Occludin) and the extracellular matrix. Reducing MMP-9 expression and activity effectively protects tight junction proteins from degradation (80). Quercetin has been demonstrated to reduce Evans blue dye extravasation, decrease BBB permeability, and improve barrier function (81).

Additionally, quercetin directly promotes tight junction protein expression. Research indicates that quercetin administration leads to substantial upregulation of critical tight junction proteins, such as zonula occluden-1 (ZO-1) and Claudin-5, in ischemic brain tissue, reinforcing intercellular connections and reducing BBB permeability (82, 83). The Wnt/*β*-catenin signaling pathway plays a pivotal role in this protective process (84). Activation of the Wnt/β-catenin pathway stabilizes β-catenin protein, promoting its nuclear translocation and regulating downstream target genes, including ZO-1 and Claudin-5 (77). Research has demonstrated that in a rat model of cerebral ischemia–reperfusion injury, quercetin enhances BBB functionality by stimulating the Wnt/*β*-catenin signaling pathway, thereby increasing ZO-1, Claudin-5, and β-catenin expression.

### 2.2 Neuroprotective effects

Beyond fortifying the vascular barrier, quercetin can also traverse the damaged BBB, albeit with limited efficiency, to exert direct effects on neurons and glial cells within the brain parenchyma, manifesting multidimensional neuroprotective effects (85).

#### 2.2.1 Suppression of neuronal apoptosis and programmed cell death

Neuronal apoptosis represents one of the primary forms of cell death in the ischemic penumbra (86). Quercetin effectively inhibits this process by regulating multiple key survival and death signaling pathways (86). Among these, the PI3K/Akt pathway is a prototypical pro-survival pathway (87). Brain-derived neurotrophic factor (BDNF) binds to its receptor, TrkB, activating downstream PI3K/Akt signaling (88). Activated Akt phosphorylates and inhibits multiple pro-apoptotic proteins while simultaneously activating anti-apoptotic proteins. Studies have demonstrated that quercetin significantly upregulates BDNF expression in ischemic brain regions, associated with activation of the BDNF–TrkB-PI3K/Akt signaling pathway. This effect inhibits neuronal apoptosis and reduces cerebral infarction volume.

Furthermore, quercetin modulates the thioredoxin system. Under oxidative stress conditions, apoptosis signal-regulating kinase 1 (ASK1) dissociates from its inhibitory protein, thioredoxin (Trx), resulting in its activation and initiating the downstream JNK/p38 MAPK apoptotic pathway (89). Research indicates that quercetin enhances Trx expression, thereby increasing its binding to ASK1 and impeding ASK1-mediated apoptotic signaling, thus exerting neuroprotective effects (90).

#### 2.2.2 Restoration of microglial function to modulate neuroinflammation

Microglia, resident immune cells of the central nervous system, are rapidly activated following brain injury and exert dual effects. On one hand, classically activated M1 microglia release substantial amounts of proinflammatory factors (e.g., TNF-*α* and IL-1*β*), exacerbating neurotoxicity. Conversely, alternatively activated M2 microglia secrete anti-inflammatory factors, such as IL-10 and TGF-β, along with neurotrophic factors, promoting tissue repair and nerve regeneration. Consequently, promoting microglial transformation from the pro-inflammatory M1 state to the anti-inflammatory M2 state is identified as a pivotal therapeutic approach for addressing neuroinflammation (91).

Quercetin exhibits precise regulatory capabilities in this regard. Research has demonstrated that in cerebral ischemia models, quercetin substantially inhibits M1 polarization of microglia, characterized by reduced expression of M1 markers such as iNOS and CD86, and enhanced conversion to M2 with increased expression of M2 markers, including Arg-1 and CD206 (92). This mechanism is partly attributable to inhibition of the TLR4/NF-κB pathway. Further research suggests that quercetin may facilitate deacetylation of high-mobility group box protein B1 (HMGB1) through SIRT1-mediated mechanisms, inhibiting HMGB1 release from the nucleus to the extracellular space, thereby blocking its role as a key pro-inflammatory signaling molecule that activates microglia (93). This approach ultimately achieves balanced regulation of microglial polarization. By modulating microglia and reducing their production of neurotoxic substances, quercetin creates a microenvironment more conducive to the survival and repair of damaged neurons (94).

#### 2.2.3 Promotion of neural recovery and synaptic plasticity

The protective effects of quercetin extend beyond mitigating acute brain injury to encompassing promotion of long-term neurological recovery. In various animal models of cerebral ischemia, quercetin administration resulted in substantial enhancement of neurological function scores, Morris water maze test performance (assessing learning and memory), and rotarod test performance (evaluating motor coordination). These functional enhancements are closely associated with its impact on synaptic plasticity.

Synaptic plasticity, particularly long-term potentiation (LTP), serves as the cellular basis of learning and memory. Upregulating proteins involved in synaptogenesis (e.g., synaptophysin and synapsin) promotes neural network remodeling, thereby enhancing synaptic plasticity. Cerebral ischemia induces substantial impairment in LTP within critical brain regions such as the hippocampus (95). Electrophysiological studies demonstrate that quercetin exhibits partial reversal of LTP suppression in CA1 pyramidal neurons of ischemic rats (96), suggesting potential for maintenance or restoration of synaptic transmission efficacy. The precise molecular mechanisms underlying this phenomenon are not yet fully elucidated; however, they may involve regulation of voltage-gated sodium channels and preservation of glutamatergic synaptic transmission (97).

### 2.3 Calcium homeostasis and mitochondrial protection

Calcium overload has been identified as a pivotal mechanism in the pathophysiology of cerebral ischemic injury (98). Quercetin maintains intracellular calcium ion balance by upregulating expression of calcium-buffering proteins, including calbindin and parvalbumin (32). The mechanisms are as follows: first, enhanced calbindin and parvalbumin expression allows these low-molecular-weight calcium-binding proteins to absorb intracellular calcium overload, preventing excessive activation of calcium-dependent proteases such as calpain (99, 100). Additionally, quercetin reduces NMDA receptor-mediated calcium influx, exerting an indirect effect on pathological calcium influx through inhibition of glutamate excitotoxicity (101).

Regarding mitochondrial protection, quercetin stabilizes mitochondrial membrane potential, enhances electron transport chain activity, and increases ATP synthesis, thereby ensuring neuronal energy supply. This phenomenon is intimately linked to activation of the AMP-activated protein kinase signaling pathway, which plays a pivotal role in cellular energy sensing (102).

### 2.4 Promotion of angiogenesis and remodeling

In the subacute and chronic phases of ischemic injury, angiogenesis and vascular remodeling are critical for restoring blood supply, salvaging ischemic penumbra tissue, and promoting functional recovery (103). Preliminary research indicates potential involvement of quercetin in this process. For instance, in models of peripheral ischemia, quercetin promotes endothelial cell survival, migration, and lumen formation, thereby increasing capillary density in ischemic tissue (104). However, it is crucial to acknowledge the context-dependent nature of quercetin’s role in angiogenesis, as it frequently exhibits anti-angiogenic properties in tumor studies. Consequently, further direct and in-depth investigation is required to elucidate its specific effects and molecular mechanisms on angiogenesis and remodeling in cerebral ischemia models.

### 2.5 Quercetin’s unique molecular targets and efficacy

In addition to the common signaling pathways it shares with other polyphenols, quercetin exhibits unique molecular interactions that are responsible for its superior protective efficacy in the cerebrovascular system. Systematic structure–activity relationship studies have shed light on the molecular basis of quercetin’s exceptional activity. An investigation into flavonoids revealed that optimal cerebrovascular protection requires (105): The 3′,4′-catechol structure in the B-ring is responsible for potent antioxidant activity and metal chelation, while the 2,3-double bond in conjugation with the 4-keto function in the C-ring enables electron delocalization. Finally, the 3-OH stabilizes the molecule and enables critical hydrogen bonding interactions with target proteins. Quercetin possesses all three of these structural prerequisites; removal or modification of any one of them substantially diminishes its activity. Comparative *in vivo* studies demonstrate quercetin’s superior efficacy in reducing cerebral infarct volume compared to equimolar doses of kaempferol, luteolin or catechin in MCAO models (106). These distinctive molecular properties, combined with unique metabolic bioactivation in ischemic tissue, establish quercetin as a particularly promising candidate among dietary flavonoids for cerebrovascular protection.

In summary, the neuroprotective effects of quercetin manifest as a multi-layered, multi-target network effect. This process involves fortifying vascular defenses to limit injury propagation while directly intervening in the fate of neurons and glial cells, promoting their survival and facilitating brain neuron repair. This comprehensive protective profile renders quercetin an exceptionally attractive candidate molecule for interventions in cerebrovascular diseases.

## 3 From diet to clinical practice: real-world challenges in quercetin application

While preclinical studies have demonstrated the broad potential of quercetin, its translation from dietary or laboratory settings to clinical applications faces significant obstacles (107). These challenges primarily stem from the complexity of dietary intake, inherent pharmacokinetic limitations, and the resulting scarcity of clinical evidence (108).

### 3.1 Dietary sources and intake considerations

Quercetin is a prevalent constituent of many individuals’ daily diets (109). Primary sources include onions (particularly red onions), apples (especially the peel), berries (e.g., blueberries and cranberries), citrus fruits, leafy greens (e.g., mustard greens), legumes, nuts (e.g., peanuts), and black and green teas. Adopting a balanced dietary pattern rich in these foods (such as the Mediterranean diet) theoretically enables sustained quercetin intake.

Nevertheless, establishing a direct correlation between dietary quercetin intake and cerebrovascular health is encumbered by methodological challenges (110, 111). First, accurately quantifying individual quercetin intake from complex diets is extremely difficult, as it is influenced by numerous factors, including variety, ripeness, growing conditions, and storage methods (112). Particularly significant is the impact of cooking and processing methods on quercetin content and bioactivity (113). For instance, boiling may cause loss of poorly water-soluble quercetin glycosides, while high-temperature baking or frying may induce thermal degradation (114). However, specific data regarding the impact of various cooking methods on quercetin content and bioactivity in common foods remain limited in the scientific literature, creating a significant knowledge gap in evaluating the practical efficacy of dietary strategies (115).

Additionally, there is a paucity of high-quality epidemiological evidence definitively establishing a dose-dependent negative correlation between dietary quercetin intake and the risk of cerebrovascular diseases such as ischemic stroke. While research has indicated a potential correlation between flavonoid-rich dietary patterns and reduced cardiovascular disease risk, a comprehensive literature search did not yield prospective cohort studies specifically examining the relationship between quercetin monomer and cerebrovascular disease risk, particularly quantitative studies providing precise risk ratios and confidence intervals (116, 117). This evidence gap renders the establishment of a definitive daily recommended quercetin intake for cerebrovascular disease prevention challenging based on current data.

### 3.2 The “delivery gap”: the bottleneck between bioavailability and the blood–brain barrier

Despite administration of high-purity quercetin via supplements, its bioavailability in the human body remains significantly lower than anticipated. Its inherent poor pharmacokinetic properties constitute a core bottleneck in clinical translation, known as the “delivery gap.”

#### 3.2.1 Low oral bioavailability

Quercetin demonstrates suboptimal water solubility, impeding its dissolution and absorption within the gastrointestinal tract. More crucially, it undergoes extensive and rapid first-pass metabolism in intestinal epithelial cells and the liver. The human body primarily converts it rapidly via Phase II metabolic enzymes into glucuronide, sulfated, and methylated derivatives. These metabolites generally exhibit reduced biological activity compared to the parent compound and are rapidly eliminated by the kidneys. Collectively, the absolute bioavailability of orally administered quercetin is generally below 6%, meaning the vast majority of ingested quercetin is metabolically inactivated and excreted before entering systemic circulation (118).

#### 3.2.2 Difficulty crossing the BBB

Importantly, even when minimal quantities of quercetin and its metabolites enter the bloodstream, they must successfully navigate the BBB to reach brain targets (116). In healthy individuals, the BBB exhibits a dense structure and expresses multiple efflux transporters (e.g., P-glycoprotein), which actively pump quercetin-like exogenous substances out of endothelial cells, preventing their entry into brain parenchyma (119). Consequently, conventionally administered quercetin (via oral or intravenous routes) achieves extremely low concentrations in brain tissue, likely failing to reach levels required to produce significant biological effects observed in *in vitro* experiments (120). This “delivery gap” plausibly explains why pronounced therapeutic effects observed in animal models (often using high-dose administration methods such as intraperitoneal injection) are difficult to replicate in humans.

### 3.3 Clinical research “vacuum zones”: evidence gaps and translation challenges

Pharmacokinetic barriers significantly impede clinical research progress. Despite extensive preclinical literature on quercetin, no large-scale, randomized, controlled Phase II or III clinical trials have been published evaluating its efficacy in treating acute ischemic stroke, secondary stroke prevention, vascular cognitive impairment, or post-stroke cognitive rehabilitation.

This “clinical research vacuum” signifies a dearth of evidence-based responses to critical inquiries: Efficacy: Can quercetin enhance neurological outcomes in stroke patients? Can it decelerate vascular cognitive impairment progression? What are the alterations in pertinent clinical endpoints, such as cognitive test scores or functional recovery scales? Dose–response relationship: What is the optimal therapeutic dose? Is there an effective dose window? Safety: What is the spectrum of adverse events in cerebrovascular disease patients during long-term or high-dose use? What is the maximum tolerated dose? Pharmacokinetic parameters: What are the specific parameters for absorption, distribution, metabolism, and excretion in patients? What are the actual drug concentrations achievable in cerebrospinal fluid or brain tissue?

Absent this high-level clinical evidence, discourse regarding quercetin’s potential as a treatment for cerebrovascular disease remains largely speculative, impeding regulatory approval and integration into clinical guidelines. Consequently, quercetin’s translational progress has stalled at a critical stage—namely, the transition from a “promising compound” to an “effective drug.”

### 3.4 Dietary physiological concentrations versus experimental pharmacological doses

A fundamental limitation of translating quercetin research into clinical practice is the substantial discrepancy between the concentrations that can be achieved physiologically and those that demonstrate robust protective effects in experimental systems. Following the consumption of quercetin-rich foods or standard supplements, the peak plasma concentration of total quercetin is only 0.2–2.3 μmol/L, with the free aglycone representing less than 5% of this total (121). Brain tissue concentrations are estimated to be 10–15% of plasma levels, resulting in cerebral quercetin concentrations of approximately 0.02–0.35 μmol/L in realistic dietary scenarios (122, 123). By contrast, animal studies demonstrating significant cerebrovascular protection usually employ intraperitoneal doses that produce peak plasma concentrations of 30–100 μmol/L, which is tens of times higher than the level achieved through oral administration (124).

Adding to the difficulty of putting this into practice, quercetin undergoes extensive phase II metabolism, with glucuronide, sulfate and methylated conjugates accounting for over 95% of circulating quercetin-related compounds. Comparative studies reveal that major metabolites, such as quercetin-3-O-glucuronide, have only 15–30% of the aglycone’s direct antioxidant capacity and significantly reduced NF-κB inhibitory efficacy (125). However, emerging evidence suggests that metabolites may retain activity through alternative mechanisms, including the inhibition of NADPH oxidase, and demonstrate superior BBB penetration. Subsequent intracellular deconjugation then regenerates the active aglycone (126).

These pharmacokinetic realities necessitate the cautious interpretation of preclinical data and suggest that the beneficial effects of dietary quercetin likely operate through mechanisms that are distinct from those elucidated in high-dose experimental paradigms. These mechanisms could potentially involve chronic, low-level anti-inflammatory effects, epigenetic modifications or modulation of the gut microbiome, rather than acute, pharmacological neuroprotection. Future research should prioritize physiologically relevant concentration ranges and investigate whether, despite their reduced direct potency, quercetin metabolites contribute meaningfully to cerebrovascular health through cumulative chronic exposure.

## 4 Future strategy: an innovation pathway to bridge the gap

To address the formidable challenges in the clinical translation of quercetin, future research must pursue a dual-pronged approach. On one hand, advanced formulation technologies must be leveraged to overcome the “delivery gap.” On the other hand, rigorous clinical trials must be conducted to obtain conclusive evidence based on improved delivery systems.

### 4.1 Strategies for enhancing the bioavailability of quercetin

To overcome the inherent pharmacokinetic limitations of quercetin, researchers are actively exploring delivery strategies across multiple medical fields with the objective of enhancing bioavailability. These strategies include: Enzymatic modification of isoquercitrin: Polysaccharides are attached to quercetin through natural enzymatic processes (127, 128). This approach has been demonstrated to increase peak plasma concentrations by up to 40-fold, with a time to peak concentration of just 15 min (127). Furthermore, the water-soluble form of quercetin exhibits significantly stronger biological activity than original quercetin. Liposomal encapsulation: Encapsulating quercetin within spherical structures composed of phospholipids that mimic cell membranes enhances bioavailability by a factor of 50, achieving equivalent or higher plasma exposure levels at doses one-fifth of those required by conventional quercetin (129). Quercetin derivatives: Glucoside conjugates, particularly the 3-O-glucoside derived from onions, demonstrate the highest bioavailability in humans.

In the domain of nanomedicine, the utilization of nanocarriers for drug delivery is regarded as the most promising strategy to address the “delivery gap.” The fundamental principle underlying this approach entails modifying quercetin’s physicochemical characteristics and *in vivo* behavior through encapsulation or conjugation to nanoparticles (130). Multiple nanodelivery systems have been employed in quercetin research, substantiating their potential: (1) Liposomes and polymeric nanoparticles: As conventional nanocarriers, encapsulating hydrophobic quercetin within a lipid bilayer or polymeric core enhances water solubility and stability, protects against premature metabolic degradation, and prolongs circulation time (131). (2) Exosomes: Exosome-based nanovesicles have emerged as a promising platform for intercellular communication, exhibiting low immunogenicity and inherent trans-biological barrier capabilities. Loading quercetin into engineered vesicles holds promise for more efficient and safe targeted delivery (132, 133). Functionalization and targeted modification: To achieve precise delivery, specific targeting molecules can be modified onto nanocarrier surfaces, enabling active recognition and binding to specific receptors on brain endothelial cells or neurons (134), thereby achieving active brain targeting. For instance, a study developed a quercetin conjugate combining a mitochondrial-targeting short peptide with a CD44 receptor-targeting hyaluronic acid (135). This system exhibited triple-targeting potential, significantly enhancing neuroprotective effects. (3) Smart responsive nanoscale systems: More advanced designs involve “smart” nanocarriers, such as ROS-responsive nanoparticles engineered to respond to microenvironmental features in ischemic brain regions (e.g., elevated reactive oxygen species levels). These particles maintain stability within healthy tissues but undergo structural alterations upon reaching the lesion site, enabling on-demand drug release, thereby enhancing efficacy and reducing side effects (136). Similarly, Jian et al. (137) developed Quercetin@TPP-ROS-Lips, which are based on the anti-inflammatory and antioxidant properties of quercetin, as well as the ROS-responsive degradation characteristics of dopamine. The Quercetin@TPP-ROS-Lips were formed through the oxidatively self-assembly of dopamine to encapsulate quercetin, with rabies virus glycoprotein coated on the surface. This significantly enhanced the diffusion stability of quercetin, exhibiting ROS-responsive degradation properties. The Quercetin@TPP-ROS-Lips effectively reduced oxidative damage in SH-SY5Y cells and induced glial cell polarization toward an anti-inflammatory (M2) phenotype. Further *in vivo* studies demonstrated that Quercetin@TPP-ROS-Lips reduced neuronal apoptosis and significantly improved neurological deficits in a rat model of middle cerebral artery occlusion. Similarly, Zhao et al. (138) encapsulated quercetin within ROS-responsive, mitochondria-targeted liposomes to achieve at least 14 days of sustained quercetin therapy for ischemia-reperfusion injury. (4) Chemical modification: Beyond nanoparticle encapsulation, chemical modification of the quercetin molecule represents a promising avenue. For instance, glycosylation modifications enhance quercetin’s water solubility, bioavailability, and brain targeting capacity while amplifying neuroprotective effects (139).

While these advanced delivery systems have shown encouraging results in preclinical models (e.g., significantly increased intracerebral drug concentrations and more effective reduction of infarct volume), recent studies frequently lack standardized reporting of key pharmacokinetic parameters, hindering cross-study comparisons. Establishing standardized evaluation systems is imperative for selecting optimal clinical candidate formulations in the future.

### 4.2 Precisely designed clinical trials: the key to translating theory into practice

Following procurement of advanced formulations capable of ensuring sufficient brain exposure, the subsequent step involves conducting rigorously designed and properly executed clinical trials. This is not only the sole pathway to validate quercetin efficacy but also an essential step in assessing patient safety. Future clinical trial designs should consider the following aspects: (1) Selecting appropriate formulations: Priority should be given to nanoformulations or chemically modified compounds that have demonstrated optimal brain targeting efficiency and safety in preclinical studies, as opposed to conventional quercetin supplements. (2) Defining target populations: Given the neuroprotective time window, quercetin formulations may be more appropriate as adjunctive therapies to existing standard treatments in acute stroke care. However, its potential for secondary stroke prevention and post-stroke cognitive rehabilitation is greater, owing to its anti-inflammatory, antioxidant, and neuroplasticity-promoting properties. Consequently, there is a compelling rationale for prioritizing long-term, multicenter randomized controlled trials focusing on these two populations. (3) Scientifically defining endpoints: Efficacy endpoints should extend beyond imaging-based infarct volume to focus on functionally meaningful patient outcomes, such as modified Rankin Scale scores, activities of daily living assessments, and standardized cognitive function scales (140, 141). (4) Strengthening pharmacokinetic and pharmacodynamic research: Concurrent pharmacokinetic and pharmacodynamic studies are imperative in clinical trials. Monitoring drug concentrations in plasma and cerebrospinal fluid, with subsequent correlation to biomarkers and clinical efficacy, will facilitate exploration of dose–response relationships and provide evidence for determining optimal dosing regimens.

Whether and how quercetin can be used to treat human cerebrovascular diseases can only be definitively answered through high-quality clinical research. This would truly achieve its transformation from a dietary component to a clinical drug.

## 5 Conclusion and outlook

Quercetin, a naturally occurring polyphenol, has been the subject of divergent research findings in the domain of cerebrovascular diseases. On one hand, extensive preclinical studies have confirmed its multifaceted protective potential through synergistic regulation of antioxidant, anti-inflammatory, and anti-apoptotic pathways, demonstrating comprehensive effects ranging from vascular endothelial repair to direct neuroprotection. Conversely, its remarkably low bioavailability and the formidable blood–brain barrier pose substantial obstacles to clinical translation, resulting in a paucity of high-level clinical evidence and preventing its theoretical potential from translating into tangible clinical benefits.

To advance quercetin application, a dual-track strategy is imperative. At the public health level, efforts should encourage and promote dietary patterns rich in flavonoids such as quercetin. While the preventive effects of these diets on specific diseases require more precise epidemiological data, the overall cardiovascular health benefits of such diets are widely recognized, aligning with the principles of low-risk, broad-benefit preventive medicine. In the domain of drug development, the primary objective entails synthesizing innovative formulations capable of effectively and safely delivering quercetin to the brain. This endeavor necessitates integrating advanced tools, including nanotechnology and medicinal chemistry, to facilitate cutting-edge solution development. Initiating and completing long-awaited large-scale clinical trials is contingent upon ensuring drug accessibility to the target site.

In summary, the full potential of quercetin in preventing and treating cerebrovascular diseases remains to be fully realized. This approach synthesizes the dietary principles of traditional Chinese herbal medicine, offering a compelling foundation for developing precision drugs in the future. The eventual clinical fate of quercetin is contingent upon our ability to successfully bridge the existing bioavailability gap through technological innovation. Moreover, it is imperative that rigorous scientific evidences guide its transition from a dietary supplement to a clinical application.
